# *N*′-[(1*E*)-1-(4-Chloro­phen­yl)ethyl­idene]formohydrazide

**DOI:** 10.1107/S1600536809037143

**Published:** 2009-09-19

**Authors:** Zahid Shafiq, Muhammad Yaqub, M. Nawaz Tahir, Mian Hasnain Nawaz, M. Saeed Iqbal

**Affiliations:** a Department of Chemistry, Bahauddin Zakariya University, Multan-60800, Pakistan; b Department of Physics, University of Sargodha, Sargodha, Pakistan; c Department of Chemistry, Government College University, Lahore, Pakistan

## Abstract

The structure of the title compound, C_9_H_9_ClN_2_O, consists of centrosymmetric dimers due to inter­molecular N—H⋯O hydrogen bonding, forming *R*_2_^2^(8) ring motifs. The dihedral angle between the *p*-chloro­phenyl unit and the remaining heavy-atom group is 6.77 (17)°.

## Related literature

For hydrogen-bond motifs, see: Bernstein *et al.* (1995 ▶). For a related structure, see: Guo (2007 ▶).
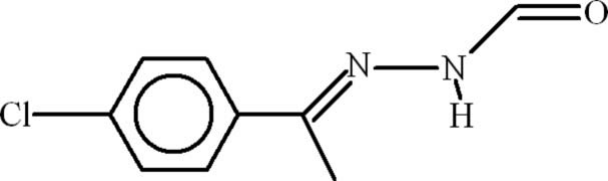


## Experimental

### 

#### Crystal data

C_9_H_9_ClN_2_O
*M* *_r_* = 196.63
Monoclinic, 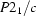

*a* = 5.9373 (5) Å
*b* = 6.2178 (4) Å
*c* = 25.3495 (18) Å
β = 93.900 (4)°
*V* = 933.66 (12) Å^3^
*Z* = 4
Mo *K*α radiation
μ = 0.37 mm^−1^
*T* = 296 K
0.25 × 0.22 × 0.18 mm

#### Data collection

Bruker Kappa APEXII CCD diffractometer
Absorption correction: multi-scan (*SADABS*; Bruker, 2005 ▶) *T*_min_ = 0.914, *T*_max_ = 0.940
9690 measured reflections
2311 independent reflections
1426 reflections with *I* > 2σ(*I*)
*R*_int_ = 0.025

#### Refinement

*R*[*F* ^2^ > 2σ(*F* ^2^)] = 0.050
*wR*(*F* ^2^) = 0.150
*S* = 1.05
2311 reflections
119 parameters
H-atom parameters constrained
Δρ_max_ = 0.26 e Å^−3^
Δρ_min_ = −0.20 e Å^−3^

### 

Data collection: *APEX2* (Bruker, 2007 ▶); cell refinement: *SAINT* (Bruker, 2007 ▶); data reduction: *SAINT*; program(s) used to solve structure: *SHELXS97* (Sheldrick, 2008 ▶); program(s) used to refine structure: *SHELXL97* (Sheldrick, 2008 ▶); molecular graphics: *ORTEP-3 for Windows* (Farrugia, 1997 ▶) and *PLATON* (Spek, 2009 ▶); software used to prepare material for publication: *WinGX* (Farrugia, 1999 ▶) and *PLATON*.

## Supplementary Material

Crystal structure: contains datablocks global, I. DOI: 10.1107/S1600536809037143/bq2157sup1.cif

Structure factors: contains datablocks I. DOI: 10.1107/S1600536809037143/bq2157Isup2.hkl

Additional supplementary materials:  crystallographic information; 3D view; checkCIF report

## Figures and Tables

**Table 1:** Hydrogen-bond geometry (Å, °)

*D*—H⋯*A*	*D*—H	H⋯*A*	*D*⋯*A*	*D*—H⋯*A*
N2—H2*A*⋯O1^i^	0.8600	2.0800	2.920 (3)	164.00

Symmetry code: (i) 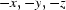.

**Fig. 1.**
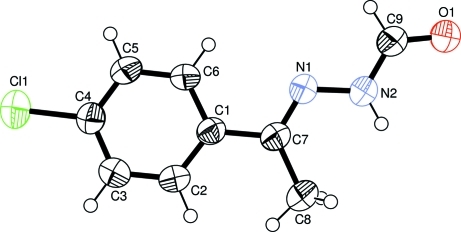
View of the title compound with the atom numbering scheme. The thermal ellipsoids are drawn at the 50% probability level.

**Fig. 2.**
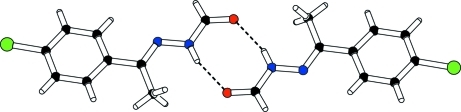
The partial packing (PLATON; Spek, 2009) which shows that molecules are dimerized and form ring motifs.

## supplementary crystallographic information

### Comment

Schiff bases are important intermediates in a number of enzymatic reactions involving interaction of an enzyme with an amino or a carbonyl group of the substrate. The title compound (I, Fig. 1), has been prepared as a derivative.

The crystal structures of *N*'-(1-(4-Chlorophenyl)ethylidene)propionohydrazide (Guo, 2007) has been published which differs from the title compound (I) due to the attachment of ethyl moiety instead of H-atom with the carbonyl group.

In the title compound, due to intermolecular H-bonding the molecules are dimerized forming 8-membered *R*_2_^2^(8) ring motifs (Table 1, Fig. 2) (Bernstein *et al.*, 1995). The *p*-Clorophenyl moiety A (C1—C6, Cl1) and the remaining heavy atoms group B (C8, C7, N1, N2 C9, O1) are almost planar with r.m.s. deviations of 0.007 and 0.009 Å, respectively, with a 6.77 (17)° dihedral angle between them.

### Experimental

formohydrazide (1 g, 0.017 mol) was dissolved in ethanol (10 ml) and stirred. To this solution 4-Chlororoacetophenone (2.067 ml, 0.017 mol) was added dropwise and refluxed for 30 min. During refluxing precipitates were formed and the reaction mixture was further heated for 2 h. The completion of reaction was monitored by TLC. The solution was cooled to room temperature and the crued solid was collected by suction filtration. The precipitates were washed with hot ethanol, filtered and dried. The colorless prisms of title compound (I) were obtained by crystallization of the crude material in 1,4-dioxan.

### Refinement

The H-atoms were positioned geometrically with N—H = 0.86, C—H = 0.93 and 0.96 Å for aromatic and methyl H atoms, respectively and constrained to ride on their parent atoms, with *U*_iso_(H) = xUeq(C,N), where *x* = 1.5 for methyl H and *x* = 1.2 for all other H atoms.

### Crystal data

**Table:**

C_9_H_9_ClN_2_O	*F*(000) = 408
*M**_r_* = 196.63	*D*_x_ = 1.399 Mg m^−^^3^
Monoclinic, *P*2_1_/*c*	Mo *K*α radiation, λ = 0.71073 Å
Hall symbol: -P 2ybc	Cell parameters from 1864 reflections
*a* = 5.9373 (5) Å	θ = 2.3–28.0°
*b* = 6.2178 (4) Å	µ = 0.37 mm^−^^1^
*c* = 25.3495 (18) Å	*T* = 296 K
β = 93.900 (4)°	Prismatic, colorless
*V* = 933.66 (12) Å^3^	0.25 × 0.22 × 0.18 mm
*Z* = 4	

### Data collection

**Table:**

Bruker Kappa APEXII CCD diffractometer	2311 independent reflections
Radiation source: fine-focus sealed tube	1426 reflections with *I* > 2σ(*I*)
graphite	*R*_int_ = 0.025
Detector resolution: 7.40 pixels mm^-1^	θ_max_ = 28.3°, θ_min_ = 3.2°
ω scans	*h* = −7→7
Absorption correction: multi-scan (*SADABS*; Bruker, 2005)	*k* = −8→8
*T*_min_ = 0.914, *T*_max_ = 0.940	*l* = −33→32
9690 measured reflections	

### Refinement

**Table:**

Refinement on *F*^2^	Primary atom site location: structure-invariant direct methods
Least-squares matrix: full	Secondary atom site location: difference Fourier map
*R*[*F*^2^ > 2σ(*F*^2^)] = 0.050	Hydrogen site location: inferred from neighbouring sites
*wR*(*F*^2^) = 0.150	H-atom parameters constrained
*S* = 1.05	*w* = 1/[σ^2^(*F*_o_^2^) + (0.0631*P*)^2^ + 0.2896*P*] where *P* = (*F*_o_^2^ + 2*F*_c_^2^)/3
2311 reflections	(Δ/σ)_max_ < 0.001
119 parameters	Δρ_max_ = 0.26 e Å^−^^3^
0 restraints	Δρ_min_ = −0.20 e Å^−^^3^

### Special details

**Table:**

Geometry. Bond distances, angles *etc*. have been calculated using the rounded fractional coordinates. All su's are estimated from the variances of the (full) variance-covariance matrix. The cell e.s.d.'s are taken into account in the estimation of distances, angles and torsion angles
Refinement. Refinement of *F*^2^ against ALL reflections. The weighted *R*-factor *wR* and goodness of fit *S* are based on *F*^2^, conventional *R*-factors *R* are based on *F*, with *F* set to zero for negative *F*^2^. The threshold expression of *F*^2^ > σ(*F*^2^) is used only for calculating *R*-factors(gt) *etc*. and is not relevant to the choice of reflections for refinement. *R*-factors based on *F*^2^ are statistically about twice as large as those based on *F*, and *R*- factors based on ALL data will be even larger.

### Fractional atomic coordinates and isotropic or equivalent isotropic displacement parameters (Å^2^)

**Table:**

	*x*	*y*	*z*	*U*_iso_*/*U*_eq_	
Cl1	0.38204 (13)	1.27773 (12)	0.21878 (3)	0.0806 (3)	
O1	0.2494 (3)	0.0349 (3)	−0.03180 (7)	0.0740 (7)	
N1	0.1941 (3)	0.4376 (3)	0.06100 (7)	0.0500 (6)	
N2	0.1350 (3)	0.2578 (3)	0.03140 (7)	0.0536 (7)	
C1	0.1329 (3)	0.6969 (3)	0.12510 (8)	0.0447 (7)	
C2	0.0192 (4)	0.7732 (4)	0.16716 (9)	0.0611 (9)	
C3	0.0928 (4)	0.9511 (4)	0.19580 (10)	0.0654 (9)	
C4	0.2836 (4)	1.0560 (4)	0.18273 (9)	0.0523 (8)	
C5	0.3992 (4)	0.9858 (4)	0.14095 (10)	0.0622 (9)	
C6	0.3239 (4)	0.8092 (4)	0.11281 (9)	0.0593 (8)	
C7	0.0599 (4)	0.5018 (3)	0.09485 (8)	0.0481 (7)	
C8	−0.1579 (4)	0.3947 (4)	0.10580 (11)	0.0743 (10)	
C9	0.2786 (4)	0.1908 (4)	−0.00268 (10)	0.0623 (9)	
H2	−0.11039	0.70239	0.17636	0.0733*	
H2A	0.00968	0.19185	0.03497	0.0644*	
H3	0.01322	0.99959	0.22383	0.0785*	
H5	0.52814	1.05794	0.13180	0.0746*	
H6	0.40342	0.76308	0.08454	0.0712*	
H9	0.41191	0.26791	−0.00468	0.0747*	
H81	−0.13024	0.24613	0.11431	0.1115*	
H82	−0.26116	0.40444	0.07507	0.1115*	
H83	−0.22185	0.46470	0.13504	0.1115*	

### Atomic displacement parameters (Å^2^)

**Table:**

	*U*^11^	*U*^22^	*U*^33^	*U*^12^	*U*^13^	*U*^23^
Cl1	0.0897 (6)	0.0672 (5)	0.0858 (5)	−0.0185 (3)	0.0123 (4)	−0.0250 (3)
O1	0.0641 (12)	0.0777 (12)	0.0818 (12)	−0.0196 (9)	0.0177 (9)	−0.0322 (10)
N1	0.0503 (11)	0.0477 (10)	0.0521 (10)	−0.0062 (8)	0.0042 (8)	−0.0047 (8)
N2	0.0498 (11)	0.0522 (12)	0.0592 (12)	−0.0094 (9)	0.0059 (9)	−0.0090 (9)
C1	0.0426 (12)	0.0427 (11)	0.0491 (12)	−0.0028 (9)	0.0055 (9)	0.0032 (9)
C2	0.0555 (15)	0.0653 (15)	0.0647 (15)	−0.0166 (12)	0.0207 (11)	−0.0094 (11)
C3	0.0656 (17)	0.0681 (16)	0.0649 (15)	−0.0096 (13)	0.0224 (12)	−0.0138 (12)
C4	0.0548 (14)	0.0467 (12)	0.0553 (13)	−0.0031 (10)	0.0032 (10)	−0.0035 (9)
C5	0.0566 (15)	0.0567 (14)	0.0756 (16)	−0.0178 (11)	0.0215 (12)	−0.0066 (12)
C6	0.0558 (14)	0.0593 (14)	0.0658 (14)	−0.0143 (11)	0.0252 (11)	−0.0120 (11)
C7	0.0454 (12)	0.0467 (12)	0.0524 (12)	−0.0047 (10)	0.0048 (10)	0.0036 (9)
C8	0.0589 (16)	0.0718 (18)	0.0944 (19)	−0.0237 (13)	0.0209 (14)	−0.0215 (14)
C9	0.0531 (15)	0.0669 (16)	0.0677 (16)	−0.0143 (12)	0.0107 (12)	−0.0152 (12)

### Geometric parameters (Å, °)

**Table:**

Cl1—C4	1.733 (3)	C4—C5	1.372 (3)
O1—C9	1.224 (3)	C5—C6	1.368 (3)
N1—N2	1.379 (3)	C7—C8	1.497 (3)
N1—C7	1.274 (3)	C2—H2	0.9300
N2—C9	1.322 (3)	C3—H3	0.9300
N2—H2A	0.8600	C5—H5	0.9300
C1—C2	1.384 (3)	C6—H6	0.9300
C1—C7	1.484 (3)	C8—H81	0.9600
C1—C6	1.385 (3)	C8—H82	0.9600
C2—C3	1.378 (3)	C8—H83	0.9600
C3—C4	1.367 (3)	C9—H9	0.9300
			
Cl1···C4^i^	3.535 (2)	H2···C8	2.6200
O1···N2^ii^	2.920 (3)	H2···H83	1.9000
O1···C8^ii^	3.288 (3)	H2A···C8	2.4600
O1···C9^iii^	3.202 (3)	H2A···H81	2.2500
O1···H2A^ii^	2.0800	H2A···H82	2.3600
O1···H6^iv^	2.8300	H2A···O1^ii^	2.0800
O1···H9^iii^	2.8600	H2A···C9^ii^	3.0100
O1···H81^ii^	2.7800	H5···C8^ix^	2.9100
N2···O1^ii^	2.920 (3)	H5···H81^ix^	2.4100
N1···H6	2.4300	H6···N1	2.4300
N2···H81	2.7100	H6···O1^iv^	2.8300
N2···H82	2.8200	H6···C9^iv^	2.9100
C4···Cl1^v^	3.535 (2)	H6···H9^iv^	2.3700
C7···C9^vi^	3.537 (3)	H9···O1^iii^	2.8600
C8···O1^ii^	3.288 (3)	H9···H6^iv^	2.3700
C9···O1^iii^	3.202 (3)	H81···N2	2.7100
C9···C9^iii^	3.537 (3)	H81···C3^x^	3.0000
C9···C7^vi^	3.537 (3)	H81···H2A	2.2500
C2···H83	2.5000	H81···H5^viii^	2.4100
C3···H81^vii^	3.0000	H81···O1^ii^	2.7800
C8···H2A	2.4600	H82···N2	2.8200
C8···H5^viii^	2.9100	H82···H2A	2.3600
C8···H2	2.6200	H83···C2	2.5000
C9···H2A^ii^	3.0100	H83···H2	1.9000
C9···H6^iv^	2.9100		
			
N2—N1—C7	118.23 (18)	O1—C9—N2	124.9 (2)
N1—N2—C9	117.37 (18)	C1—C2—H2	119.00
C9—N2—H2A	121.00	C3—C2—H2	119.00
N1—N2—H2A	121.00	C2—C3—H3	120.00
C6—C1—C7	120.71 (19)	C4—C3—H3	120.00
C2—C1—C7	122.53 (18)	C4—C5—H5	120.00
C2—C1—C6	116.75 (19)	C6—C5—H5	120.00
C1—C2—C3	121.8 (2)	C1—C6—H6	119.00
C2—C3—C4	119.6 (2)	C5—C6—H6	119.00
Cl1—C4—C5	119.44 (19)	C7—C8—H81	109.00
Cl1—C4—C3	120.45 (19)	C7—C8—H82	109.00
C3—C4—C5	120.1 (2)	C7—C8—H83	109.00
C4—C5—C6	119.7 (2)	H81—C8—H82	109.00
C1—C6—C5	122.1 (2)	H81—C8—H83	109.00
N1—C7—C1	115.47 (19)	H82—C8—H83	109.00
N1—C7—C8	124.97 (19)	O1—C9—H9	118.00
C1—C7—C8	119.56 (19)	N2—C9—H9	118.00
			
C7—N1—N2—C9	−178.5 (2)	C6—C1—C2—C3	−0.5 (3)
N2—N1—C7—C8	1.0 (3)	C7—C1—C2—C3	178.1 (2)
N2—N1—C7—C1	−179.61 (17)	C6—C1—C7—C8	−174.7 (2)
N1—N2—C9—O1	−179.6 (2)	C1—C2—C3—C4	−0.2 (4)
C2—C1—C6—C5	0.6 (3)	C2—C3—C4—C5	0.8 (4)
C7—C1—C6—C5	−178.0 (2)	C2—C3—C4—Cl1	−179.02 (19)
C2—C1—C7—C8	6.7 (3)	Cl1—C4—C5—C6	179.14 (19)
C6—C1—C7—N1	5.9 (3)	C3—C4—C5—C6	−0.7 (4)
C2—C1—C7—N1	−172.7 (2)	C4—C5—C6—C1	−0.1 (4)

Symmetry codes: (i) −*x*+1, *y*+1/2, −*z*+1/2; (ii) −*x*, −*y*, −*z*; (iii) −*x*+1, −*y*, −*z*; (iv) −*x*+1, −*y*+1, −*z*; (v) −*x*+1, *y*−1/2, −*z*+1/2; (vi) −*x*, −*y*+1, −*z*; (vii) *x*, *y*+1, *z*; (viii) *x*−1, *y*−1, *z*; (ix) *x*+1, *y*+1, *z*; (x) *x*, *y*−1, *z*.

### Hydrogen-bond geometry (Å, °)

**Table:**

*D*—H···*A*	*D*—H	H···*A*	*D*···*A*	*D*—H···*A*
N2—H2A···O1^ii^	0.8600	2.0800	2.920 (3)	164.00

Symmetry codes: (ii) −*x*, −*y*, −*z*.
